# The complete chloroplast genome of *Hydrocotyle pseudoconferta* Masamune 1932 (Araliaceae)

**DOI:** 10.1080/23802359.2022.2090292

**Published:** 2022-07-04

**Authors:** Jun Wen, Wei Zhou, Bao-Cheng Wu, Hui-Min Li, Chun-Feng Song

**Affiliations:** Jiangsu Key Laboratory for the Research and Utilization of Plant Resources, Institute of Botany, Jiangsu Province and Chinese Academy of Sciences (Nanjing Botanical Garden Mem. Sun Yat-Sen), Nanjing, China

**Keywords:** Araliaceae, *Hydrocotyle*, chloroplast genome, phylogenetic analysis

## Abstract

*Hydrocotyle pseudoconferta* was an important medicinal plant. The complete plastid genome of this species was reported for the first time. The full length of the complete chloroplast genome is 153,302 bp, with a typical quadripartite organization: a large single-copy (LSC) region of 84,417 bp, a small single-copy (SSC) region of 18,767 bp, and a pair inverted repeat regions (IR_a_ and IR_b_) with 25,059 bp for each. The complete chloroplast genome of *H. pseudoconferta* encoded 133 genes, comprising 86 protein-coding genes, 37 tRNA genes, 8 rRNA genes, and 2 pseudogenes. The phylogenetic analysis suggested the closest relationship between *H. pseudoconferta* and *Hydrocotyle nepalensis*.

*Hydrocotyle pseudoconferta* Masamune 1932 is a medicinal herb species of the genus *Hydrocotyle* Tourn. ex L. The genus was formerly classified in the family Apiaceae and later transferred into Araliaceae inferred from a limited number of DNA fragments (Chandler and Plunkett 2004; Plunkett et al. 2004). In recent years, comparative analysis of the complete chloroplast genome sequences has been used as an effective tool for plant phylogeny analysis. The relationships among *Hydrocotyle*, Apiaceae, and Araliaceae may also be elucidated by the same means. Many chloroplast genomes of Apiaceae and Araliaceae have been reported, with only three species of *Hydrocotyle* included (Downie and Jansen 2015; Ge et al. 2017; Wen et al. 2021). We herein assembled and annotated the complete chloroplast genome sequence of *H. pseudoconferta* as supplementary material for further study.

Species of *H. pseudoconferta* is naturally distributed from southern China to Myanmar, and narrowly grew in wet valleys at altitudes of 800–1500 m (Sheh et al. 2005). The solitary axillary sessile umbel is the main characteristic that distinguishes this species from other *Hydrocotyle* species. Fresh leaves of *H. pseudoconferta* (Collection number: wj_2021072302) were collected from Cangnan county, Zhejiang province, China (27°27′56.18″N, 120°19′0.64″E). The voucher specimen (no. NAS00637160) was deposited in the herbarium of Nanjing Botanical Garden Mem. Sun Yat-Sen (http://www.cnbg.net, Zeng-lai Xu, 1355655293@qq.com). The total genomic DNA was extracted with a modified CTAB method (Doyle 1987) and sequenced paired-end (PE) using Illumina Novaseq platform (Illumina novaseq6000, Illumina, San Diego, CA). The raw reads were assembled using NOVOPlasty 4.3.1 (Dierckxsens et al. 2017) and then annotated using Geneious 11.1.5 (Kearse et al. 2012).

The complete chloroplast genome of *H. pseudoconferta* (GenBank accession: OK585058) is 153,302 bp in length, with 37.6% GC contents and was consisted of four regions: including two inverted repeat regions (IR_a_ and IR_b_, 25,059 bp for each) separated by a large single-copy gene region (LSC, 84,417 bp) and a small single-copy gene region (SSC, 18,767 bp). The chloroplast genome has 133 genes in total, including 86 protein-coding genes, 37 tRNA genes, 8 rRNA genes, and 2 pseudogenes.

The complete chloroplast genomes of 26 species (involving 12 Apiaceae species, 12 Araliaceae species, and 2 outgroups belonging to Torricelliaceae) were selected to reconstruct the phylogenetic position of this species. Data matrices were aligned using MAFFT v7 (Katoh and Standley 2013). A maximum-likelihood (ML) phylogenetic tree was generated based on a data matrix of a concatenation of 77 protein-coding sequences, implemented with RAxML v8 (Stamatakis 2014) under the GTR + G model for 1000 bootstrap replicates (Figure 1). The phylogenetic analysis suggested that *Hydrocotyle* was recovered as a sister group of Araliaceae, and *H. pseudoconferta* is the closest sister group of *Hydrocotyle nepalensis* Hook. 1822 within the genus. This study extends our comprehension of chloroplast genome evolution in *Hydrocotyle*.

**Figure 1.**
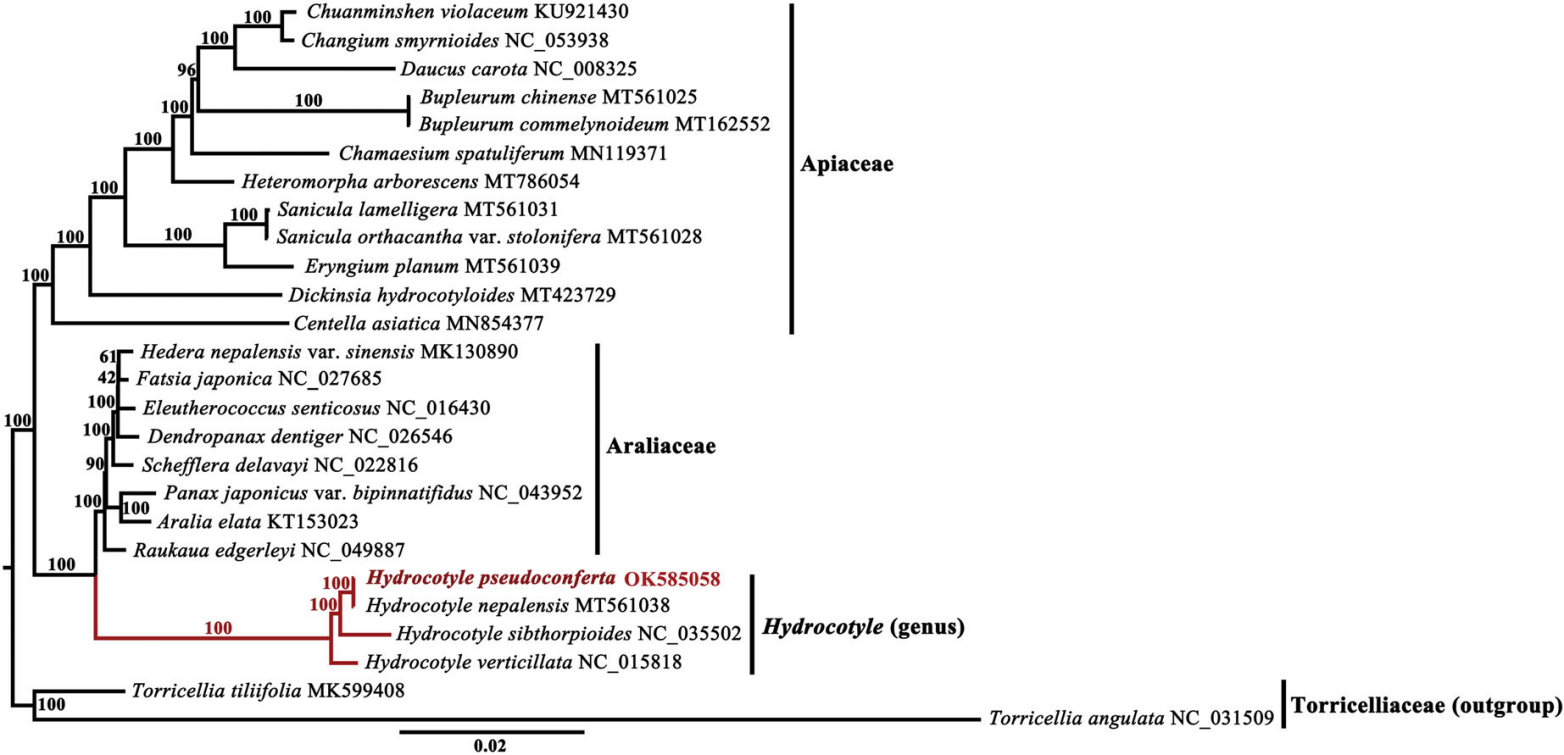
The maximum-likelihood (ML) phylogenetic tree reconstructed from protein-coding sequences of 26 complete chloroplast genomes. Numbers beside each node indicate bootstrap support values.

## Data Availability

The data that support the findings of this study are openly available in GenBank of NCBI at https://www. ncbi.nlm.nih.gov/under the Accession no. OK585058. The associated BioProject, SRA, and Bio-Sample numbers are PRJNA772920, SRR16548130, and SAMN22420039, respectively.
